# Rasburicase-Induced Methemoglobinemia

**DOI:** 10.7759/cureus.14406

**Published:** 2021-04-10

**Authors:** Moeed Ahmed, Thomas Sanchez, Selinam Norgbe, Christopher R Picking, Paul G Millner

**Affiliations:** 1 Internal Medicine, Creighton University School of Medicine, Omaha, USA

**Keywords:** rasburicase, methemoglobinemia

## Abstract

Methemoglobinemia occurs as iron in heme is oxidized to its ferric state, resulting in a decreased ability of hemoglobin to bind and release oxygen. Rasburicase is a recombinant urate-oxidase enzyme used in the prevention of tumor lysis syndrome. Methemoglobinemia can occur as a rare complication of treatment with rasburicase, primarily in patients with glucose-6-phosphate dehydrogenase (G6PD) deficiency. Methylene blue, an agent used for treating methemoglobinemia, should be avoided in patients with G6PD deficiency. In patients with G6PD deficiency, methylene blue is inadequately reduced to its active form, which then causes the methylene blue to further the oxidize the hemoglobin to methemoglobin that can result in hemolysis.

## Introduction

Rasburicase is a recombinant urate-oxidase enzyme used in the prevention of tumor lysis syndrome in patients with malignancy. Tumor lysis syndrome (TLS) is a condition that occurs when a large number of cancer cells die within a short period, releasing their contents into the blood. When chemotherapy induces cellular death, nucleic acids are broken down, with one byproduct being uric acid. Uric acid can overwhelm the proximal tubular reabsorption capacity and cause acute kidney injury due to multiple mechanisms such as intrarenal crystallization, renal vasoconstriction, and inflammation [1,2]. The aforementioned prevention of tumor lysis syndrome is accomplished by rasburicase’s ability to convert uric acid into allantoin, which is excreted by the kidneys [3]. Methemoglobinemia can occur as a rare complication of treatment with rasburicase, primarily in patients with glucose-6-phosphare dehydrogenase (G6PD) deficiency. This occurs when an oxidizing agent, such as rasburicase, dapsone, nitrates, or methylene blue, converts iron from the ferrous (Fe2+) hemoglobin state to the ferric (Fe3+) methemoglobin state [4]. This causes a physiologic left shift in the oxygen dissociation curve leading to increased oxygen binding by red blood cells and decreased oxygen delivery to cells. G6PD-deficient individuals are more susceptible to methemoglobinemia from rasburicase.

## Case presentation

A 72-year-old man who was recently diagnosed with high-grade neuroendocrine carcinoma of prostate, small cell type, presented to the hospital for evaluation of shortness of breath and abdominal pain. Initially, he was suspected to have pulmonary embolism after a ventilation/perfusion (V/Q) scan showed intermediate probability for pulmonary embolism and lower extremity duplex confirmed an acute deep vein thrombosis (DVT) of the right femoral and popliteal veins. Initially, the patient did not require supplemental oxygen. Anticoagulation was initiated, resulting in gross hematuria due to his bladder mass and underlying prostate cancer.

Given the diagnosis of neuroendocrine carcinoma of the prostate, he was started on chemotherapy with carboplatin and etoposide. Subsequent blood workup revealed a creatinine of 4.07 mg/dl, potassium level of 5.2 mmol/L (normal: 3.7-5.1 mmol/L), phosphorus level of 5.9 mg/dl (normal: 2.5-4.9 mg/dl), and a uric acid level of 11.5 mg/dl (normal: 2.6-7.2 mg/dl). Given the concern for tumor lysis syndrome and renal dysfunction, he was started on rasburicase. Two to three days after starting rasburicase, his oxygen saturation was measured between 70% and 80% on room air, confirmed with a normal tracing on the plethysmograph. There was a notable discrepancy between transcutaneous oximetry and arterial blood gas oxygen saturation. He had no shortness of breath, and a chest radiograph was unremarkable. He was placed on four liter per minute oxygen via nasal cannula, with improvement in SpO2. Lungs were clear to auscultation. Hemoglobin was stable. The methemoglobin level was found to be elevated at 5.1% (normal: 0.0%-1.5 %). There was high suspicion of rasburicase-induced methemoglobinemia; therefore, rasburicase was discontinued. Ascorbic acid and blood products were given, and the patient’s oxygen saturation subsequently improved to over 90% on room air. A G6PD level with a corresponding red blood cell level of 2.98 m/μl (normal 4.3-5.9 m/μl) was ordered and subsequently came back low at 36 (normal range: 127-427 units/trillion RBCs).

## Discussion

While the complication of rasburicase-induced methemoglobinemia is rare, occurring in fewer than 1% of patients, this complication can be life-threatening [5]. Common presenting symptoms include, but are not limited to, dyspnea, dizziness, cyanosis, oxygen saturation between 70% and 85% with a normal partial pressure of oxygen on arterial blood gas, “chocolate”-colored blood, seizures, and arrhythmias [6]. The normal range of methemoglobin level in the blood is between 0% and 2%; while levels above 20% can cause symptoms, levels above 70% can cause death [7].

G6PD deficiency increases the risk for methemoglobinemia because this enzyme reduces glutathione which in the reduced state is a cellular buffer for oxidized molecules [8]. When hemoglobin is oxidized to methemoglobin by rasburicase, the cell’s capacity to reduce methemoglobin is overwhelmed, causing the toxic substance to build up in the body and present with symptoms [9]. Patients, especially in high-risk ethnic groups, should be tested for G6PD deficiency prior to the administration of rasburicase. Some of the factors that placed our patient at a higher risk for tumor lysis syndrome is that the patient had a solid tumor, which was responsive to the chemotherapy, as well as the fact that the patient had an existing renal dysfunction that may increase the risk of this patient progressing into tumor lysis syndrome [10]. There are fewer than 28 cases with a known rasburicase-induced methemoglobinemia in patients with a G6PD deficiency, and at least two cases of patients were found who died from complications associated with their methemoglobinemia [5,11-14]. Rasburicase should not be given to patients with a G6PD deficiency because hydrogen peroxide is produced when the rasburicase coverts the uric acid to allantoin. Patients with a G6PD deficiency are not able to detoxify the hydrogen peroxide, which then starts to oxidize the hemoglobin to methemoglobin. Methylene blue, an agent used for treating methemoglobinemia, should be avoided in patients with G6PD deficiency considering that it is inadequately reduced to its active form, which can lead to hemolysis [15]. As rasburicase-induced methemoglobinemia mainly occurs in individuals with G6PD deficiency, empirical use of methylene blue should be avoided in these individuals. One of the concerns is that the G6PD level can take days to be resulted, which increases the importance of testing the patients earlier. Initial management involves discontinuation of the offending agent with subsequent options including the administration of ascorbic acid and blood administration.

## Conclusions

Low oxygen saturation in a patient who is being given rasburicase should raise suspicion for methemoglobinemia, and these patients should be treated as being G6PD-deficient until the G6PD level is resulted. Methylene should be avoided in these patients to prevent hemolysis. Allopurinol may be considered as an alternative option in these patients.
